# Tensin3 Is a Negative Regulator of Cell Migration and All Four Tensin Family Members Are Downregulated in Human Kidney Cancer

**DOI:** 10.1371/journal.pone.0004350

**Published:** 2009-02-04

**Authors:** Danuta Martuszewska, Börje Ljungberg, Martin Johansson, Göran Landberg, Cecilia Oslakovic, Björn Dahlbäck, Sassan Hafizi

**Affiliations:** 1 Department of Laboratory Medicine, Section for Clinical Chemistry, Lund University, University Hospital Malmö, Malmö, Sweden; 2 Department of Surgical and Perioperative Sciences, Umeå University, Umeå, Sweden; 3 Department of Laboratory Medicine, Section for Pathology, Lund University, University Hospital Malmö, Malmö, Sweden; Deutsches Krebsforschungszentrum, Germany

## Abstract

**Background:**

The Tensin family of intracellular proteins (Tensin1, -2, -3 and -4) are thought to act as links between the extracellular matrix and the cytoskeleton, and thereby mediate signaling for cell shape and motility. Dysregulation of Tensin expression has previously been implicated in human cancer. Here, we have for the first time evaluated the significance of all four Tensins in a study of human renal cell carcinoma (RCC), as well as probed the biological function of Tensin3.

**Principal Findings:**

Expression of Tensin2 and Tensin3 at mRNA and protein levels was largely absent in a panel of diverse human cancer cell lines. Quantitative RT-PCR analysis revealed mRNA expression of all four Tensin genes to be significantly downregulated in human kidney tumors (50–100% reduction versus normal kidney cortex; *P*<0.001). Furthermore, the mRNA expressions of Tensins mostly correlated positively with each other and negatively with tumor grade, but not tumor size. Immunohistochemical analysis revealed Tensin3 to be present in the cytoplasm of tubular epithelium in normal human kidney sections, whilst expression was weaker or absent in 41% of kidney tumors. A subset of tumor sections showed a preferential plasma membrane expression of Tensin3, which in clear cell RCC patients was correlated with longer survival. Stable expression of Tensin3 in HEK 293 cells markedly inhibited both cell migration and matrix invasion, a function independent of putative phosphatase activity in Tensin3. Conversely, siRNA knockdown of endogenous Tensin3 in human cancer cells significantly increased their migration.

**Conclusions:**

Our findings indicate that the Tensins may represent a novel group of metastasis suppressors in the kidney, the loss of which leads to greater tumor cell motility and consequent metastasis. Moreover, tumorigenesis in the human kidney may be facilitated by a general downregulation of Tensins. Therefore, anti-metastatic therapies may benefit from restoring or preserving Tensin expression in primary tumors.

## Introduction

The Tensins constitute a family of intracellular proteins that are coming to the fore as novel regulators of cell motility and growth. The Tensin family is composed of four members: Tensin1, Tensin2, Tensin3 and Tensin4 (TNS1, TNS2, TNS3, TNS4) each with discrete expression patterns in the human body [1], [2]. Based on their common domains, the Tensins have the potential to interact with the plasma membrane (C1 domain) as well as bind to tyrosines (SH2 and PTB domain) in integrins [3] and receptor tyrosine kinases (RTKs) [1].

In addition, Tensins 1-3, but not -4, contain a phosphatase (PTPase)-C2 domain pair that is homologous to that in the PTEN tumour suppressor protein [4], [5]. However, the potential lipid PTPase activity of Tensins 1–3, as well as their putative interactions with the actin cytoskeleton, remains to be determined. Tensin1 was the first member identified, and is localized to focal adhesions in cells [6]. We identified Tensin2 (also known as C1-TEN, TENC1) as a binding partner for Axl RTK through its SH2-PTB region [1]. Tensin3 has virtually the same domain organization as C1-TEN, and interacts with the EGF receptor [7]. Tensin4 is a shorter protein that is not expected to interact with the cytoskeleton, whilst it contains the SH2-PTB domains similar to the other Tensins [8].

The functional consequence of Tensin interactions suggests regulation of membrane receptor signaling that is linked to control of cytoskeletal dynamics. This therefore has implications for cell motility in the metastatic process. For example, in breast cancer cells, Tensin3 was shown to anchor integrins to the cytoskeleton, rendering the cell less motile and thereby less capable of metastasizing [9]. Conversely, stimulation by epidermal growth factor (EGF) upregulates Tensin4, which is a shorter protein that could lack actin-binding regions but which still interacts with integrins. This mobilization of Tensin4 results in its displacement of Tensin3 from the integrin, thus destabilizing the cytoskeleton and enhancing cell motility. We have reported that Tensin2 expression in kidney cells inhibits cell proliferation, survival and migration, concomitant with a selective suppression of the Akt signaling pathway [5]. Finally, an interaction apparently common to all four Tensins is that with the DLC-1 tumor suppressor protein, which may be a mediator for the potential anti-tumor effects of Tensins [10]–[12].

In the United States, 54390 new cases of renal cancer are predicted to occur in 2008, causing an estimated 13010 deaths [13]. Renal cell carcinoma (RCC) is a cancer originating from kidney epithelium and accounts for 85% of kidney cancers and associated mortality. RCC can be further subdivided into different subtypes: conventional or clear cell RCC (ccRCC), which represents the large majority of RCC cases, followed by papillary (pRCC) and chromophobe (chRCC). Also included in this study is oncocytoma, which is a non-RCC, benign tumor type. Each of these tumor types has a distinct pathogenesis. For example, two thirds of ccRCC cases are linked with a defect in the von Hippel-Lindau (VHL) tumor suppressor gene [14].

However, the epithelial cell transformation that occurs in common to these tumor types involves multiple processes, including mutations that enable conditions favorable for cancer cell survival, proliferation and migration. In addition, enhanced cancer cell motility is a principal feature in the early metastatic process. As a quarter of RCC patients present with locally invasive or metastatic disease, it is imperative to identify the factors and mechanisms that underlie the metastatic process.

The kidney is the organ where most Tensins are preferentially expressed [1], [6], [7]. Deletion of the *Tensin2* gene was shown to be behind a mouse model of nephrotic syndrome [15], and knockout mice for Tensins -1 and -3 exhibit kidney tubular cyst formation and renal failure [16], [17]. Therefore, there is a distinct need to investigate the Tensins for their expression and functions in the human kidney in both normal and disease states. The aim of the present study was to investigate the role of the entire Tensin family in human RCC.

## Methods

### Cell culture

The following human cancer cell lines were cultured and prepared for qRT-PCR and western blot analysis: breast (MCF-7, MDA-MB231), prostate (DU145, PC3, PNT-1A), colorectal adenocarcinoma (SW480), cervical carcinoma (HeLa S3), osteosarcoma (U20S), melanoma (WM9, WM266-4, WM793), non-small lung cancer (H358, H2087, H226, H727) and B cell lymphoma (U629, U2932). Various human non-cancer cell lines were also analyzed: endothelial cell line (EAhy926), dermal fibroblasts (AG52), kidney proximal tubular cells (HK-2), breast epithelial cells (MCF-10A). Cells were grown in DMEM supplemented with 10% fetal bovine serum, 20 mM glutamine, 100 U/ml penicillin and 100 µg/ml streptomycin at 37°C in a humidified atmosphere of 5% CO_2_ in air.

### Patients

The study included 260 patients with histopathologically verified renal cell carcinoma (RCC) after nephrectomy performed at the Department of Urology, Umeå University Hospital, between 1982–2003. The study was approved by the ethical committee of Umeå University and by the Institutional Review Board, and each patient participated after providing informed consent. The clinicopathological characteristics of the patients are summarized in Table 1.

**Table 1:** Clinicopathological variables for renal cell carcinoma patients in this study.

Factor	Distribution
Number of patients	260
Patient sex M/F	156/104
Mean patient age	66 (range 25–85)
Tumor size	6–250 (median 70) mm
Tumor grade 1/2/3/4	13/48/129/61
TNM stage I/II/III/IV	66/42/64/79
Tumor type ccRCC/pRCC/chRCC/oncocytoma	205/30/13/8
Tumor type ccRCC/pRCC/chRCC/oncocytoma as source of matched normal tissue	36/7/3/2
Venous invasion no/yes	157/103
Regional lymph-node involvement no/yes	65/29

Staging procedures included a physical examination, chest radiography, ultrasonography and computerized tomography of the abdomen. Tumors were staged according to the TNM classification system 2002 [18] and histopathological grading was done according to Skinner *et al.* [19]. The RCC type was defined according to the Heidelberg consensus conference [20]. Tumor size was measured on the surgical specimens and/or on computerized tomography. Tumor size varied from 0.6 cm to 25 cm (median: 7.0 cm). Venous invasion was defined as tumor invasion in major renal veins, verified microscopically in tissue slices from the renal hilum. Patient follow-up status was assessed at least yearly by routine clinical follow-up at Umeå University Hospital or by contacting patients directly. During the follow-up period, among the 260 patients, 139 had died of the disease, 61 had died from other causes, 7 were alive with disease, and 53 were alive and free of disease. Tumor and kidney cortex tissue were sampled immediately after the nephrectomy. Samples from histopathologically nonmalignant kidney cortex tissue remote from the tumor zone were also obtained from 48 patients and used for comparative evaluation.

### Electrophoresis and western blot

Cells were lysed in lysis buffer (1% NP-40, 1% deoxycholate, 5 mM EDTA, 1 mM EGTA in PBS, pH 7.4) and separated on 5% and 8% polyacrylamide SDS gels under reducing conditions and then transferred to a PVDF membrane. Membranes were blocked in 3% fish gelatin in 0.1 M Tris-HCl, pH 8.0, 1.5 M NaCl and 0.5% Tween 20 (washing buffer), probed with a primary antibody against Tensin2 (1∶1000 dilution) and Tensin3 (1∶1000), respectively, for 1 h, washed with washing buffer followed by incubation for 1 h with the secondary antibody linked to either horseradish peroxidase (HRP) or alkaline phosphatase (AP). Afterwards, the membranes were visualized by a chemiluminescence method (HRP) or by the reduction of the 4-nitroblue tetrazolium salt (NBT) in the presence of 5-bromo-4 chloro-3 indol-phosphate (BCIP) in 0.1 M Tris-HCl, 0.05 M MgCl_2_, 0.1 M NaCl, pH 9.5 (AP).

Antibodies used for detection of Tensin2 and Tensin3 were both in-house rabbit polyclonal antibodies generated against peptides corresponding to the C terminus of each protein. The crude antisera were purified sequentially, first by affinity chromatography on protein A and G sepharose columns and subsequent further affinity purification using the immobilized peptide antigens, as previously described for anti-Tensin2 [5]. Anti-Tensin3 antibodies were tested for specificity using cell lysates expressing full-length recombinant Tensin3, as well as by blocking with the Tensin3 C-terminal peptide antigen (Figure S1). A commercial rabbit polyclonal anti-Tensin3 antibody (Sigma) was employed for western blots of stably transfected Tensin3 cell lysates. Optimal dilutions were determined for western blotting.

### Real-time quantitative reverse transcription-PCR (qRT-PCR)

For qRT-PCR analysis of kidney cancer patient samples, the viable area of each tumor tissue was used to obtain high-quality RNA, extracted using TRIzol reagent (Invitrogen, Stockholm, Sweden). From cultured cells, total RNA was extracted using CellDirect™ One-Step qRT-PCR Kit (Invitrogen, Lidingö, Sweden). RNA concentrations were quantified by spectrophotometric measurement at 260 nm wavelength (DU640 spectrophotometer, Beckman Coulter, Bromma, Sweden) and RNA integrity was verified by ethidium bromide staining of 28S and 18S rRNA after agarose gel electrophoresis.

One-step multiplex real-time quantitative RT-PCR was employed, where amplification of both the gene of interest and endogenous control gene were performed together in the same reaction tube. Gene-specific primers and Taqman probes were purchased from Applied Biosystems (Applera Sweden, Stockholm, Sweden). For each sample, 40 ng of total RNA was mixed with a reverse transcriptase and polymerase enzyme master mix, and TaqMan® MGB probes and primers were added to this mix to a final volume of 25 µl. The qRT-PCR reaction was performed in an ABI PRISM 7900HT system (Applera Sweden, Stockholm, Sweden), employing the following cycling conditions: reverse transcription for 15 min at 50°C; 2 min at 95°C, and 40 cycles of: 95°C for 15 sec and 60°C for 60 sec. Each measurement was performed in duplicate and the threshold cycle (C_t_) was determined for each amplification curve.

In order to generate standard curves for quantitative analysis of each gene, a cell line was chosen that was a reliable and verified source of mRNA for that particular gene. RNA template was omitted in negative controls, and a standard curve from serial dilutions of total RNA from the relevant cell line was obtained for all runs and each gene of interest. For each sample, the mean of duplicate Ct values was converted to ng RNA from linear regression analysis using a relative standard curve method using Excel package (Microsoft, Redmond, Washington). Each determined RNA value was then normalized for the endogenous reference gene content of the same sample. From a screen of 11 candidate housekeeping genes, the human β2-microglobulin (*B2M*) and glyceraldehyde-3-phosphate dehydrogenase (*GAPDH*) genes were empirically determined by us to be the most robust endogenous control genes for human kidney tissue and human cell lines, respectively (data not shown). Results obtained were arbitrarily expressed as ng Tensin RNA/ng endogenous control RNA.

### Northern blot analysis

Human multiple tissue Northern (MTN) blots (BD Biosciences, Stockholm, Sweden), containing 2 µg poly A+ RNA each from eight cancer cell lines, were probed for expression of Tensin2 mRNA. A 1.6 kb 3-terminal cDNA sequence common to all splice variants of Tensin2 was used as ^32^P-labelled probe. The cDNA sequence for human β-actin was used as a control probe. Radiolabeling with [^32^P]dCTP was achieved through the Rediprime II DNA labeling system (GE Healthcare, Uppsala, Sweden). Hybridization was carried out overnight at 42°C in ULTRAhyb hybridization buffer (Ambion, Stockholm, Sweden), followed by high stringency washes. Membranes were exposed to a Phosphor-Imager screen at least overnight prior to vizualization.

### Immunohistochemical analysis of Tensin3 in human kidney tumors

For the construction of tissue microarrays (TMAs), RCC sections were collected as described above, and screened by primary evaluation of hematoxylin/eosin-stained slides before TMA preparation as described previously [21]. Briefly, two 0.6 mm diameter tissue cores were collected from each tumour block and arranged in a recipient block using a manual tissue arrayer (Beecher Inc., Sun Prairie, WI). TMA sections at 4 µm thickness were deparaffinized and microwave treated for antigen retrieval according to standard procedures. The quality and robustness of the RCC TMA has been tested for other factors and reported by us previously [22]. The rabbit polyclonal anti-Tensin3 antibody used for TMA immunostaining is described above and was verified for specificity from analysis of mock- and Tensin3-transfected cells as well as by antigen blocking (Figure S1). For immunostaining, the antibody was used at an optimally determined dilution of 1∶300. Detection was performed with horseradish peroxidase using the Dako EnVision and TechMate 500 systems, and counterstaining with hematoxylin and eosin. TMA sections from 148 patients were analyzed and staining was grouped into no, low, medium, or high expression of Tensin3, as well as additionally for presence or absence of staining at the plasma membrane (PM). All TMAs were evaluated independently by two observers.

### Generation of recombinant Tensin3-expressing cells

The complete cDNA sequence for Tensin3 (1409 amino acids; NCBI accession no. NM_022748) was cloned into pcDNA3 mammalian expression vector (Gibco Invitrogen, Lidingö, Sweden) for stable expression in human embryonic kidney (HEK) 293 cells, as previously described [5]. Plasmid-bearing cells were selected for growth in the presence of the neomycin analog G418 (400 µg/ml). In parallel, mock-transfected cell clones were also developed bearing empty vector only, as well as mutant clones containing a putative PTPase-dead mutant Tensin3 (Tns3 Mut) cDNA. This mutant was generated in the putative PTPase active site of Tensin3 with cysteine at position 107 substituted for a serine. Mutation of the wildtype Tensin3 cDNA in pcDNA3 vector was performed by QuikChange site-directed mutagenesis (Stratagene, Amsterdam, The Netherlands) according to the manufacturer's instructions, using the following primers (switched nucleotide underlined): 5′-CATGTGGTCGTCATTCACAGCAGGGGCGGGAAAGGAC-3′ (sense) and 5′-GTCCTTTCCCGCCCCTGCTGTGAATGACGACCACATG-3′ (antisense).

### Cell proliferation assays

Cell proliferation was assessed in two ways. The first was measurement of mitochondrial activity as a reflection of viable cell number, as previously described [23]. Briefly, separate clones of stably transfected mock 293 cells, wildtype Tensin3 cells (Tns3 wt) and Tensin3 PTPase mutant cells (Tns3 Mut) were seeded sparsely at 0.5×10^4^ cells per well in a 96-well microtiter plate, in medium containing 0.5% serum (day −1). The next day (day 0), the medium was replaced with that containing 5% serum, and cells were further incubated for up to 7 days. On each successive day, the numbers of viable cells were measured by their conversion during 3 h of the tetrazolium compound [3-(4,5-dimethylthiazol-2-yl)-5-(3-carboxymethoxyphenyl)-2-(4-sulfophenyl)-2H-tetrazolium (MTS, Sigma) into a soluble formazan product that was measured spectrophotometrically at 490 nm. Quadruplicate wells were used for each measurement.

The second method for cell proliferation was quantitation of absolute cell numbers to generate a growth curve over 5 days. Separate clones of stably transfected mock 293 cells, wildtype Tensin3 cells (Tns3 wt) and Tensin3 PTPase mutant cells (Tns3 Mut) were seeded at 1×10^4^ cells per well in a 48-well plate, in medium containing 0.5% serum (day −1). The next day (day 0), the medium was replaced with that containing 5% serum, and cells were incubated for a further 5 days. On each successive day, cell number per well was determined by trypsinization of cells from the wells and counting in a hemocytometer chamber. For each clone, the cell number was determined from the mean count from nine fields. In both experiments, statistical differences between cell types on each day were determined by ANOVA with Bonferroni post-hoc correction.

### Cell migration and cell matrix invasion assays

A modified Boyden chamber assay system was used to determine migration/haptotaxis of Tensin3–transfected cells as previously described [5]. Briefly, cell culture inserts with 8 µm pore size membranes (Nunclon, Roskilde, Denmark) were precoated with fibronectin (10 µg/ml; Sigma, Stockholm, Sweden) on the underside. Inserts were placed into wells of 24-well plates containing complete growth medium, and 10^5^ cells were seeded into each insert interior in complete medium. Duplicate inserts were used for each separate cell line analyzed. Cells were allowed to migrate at 37°C in a cell culture incubator for 18 h. Afterwards, inserts were removed and medium inside aspirated. All cells left on the upper surface of the membrane were wiped away and insert membranes were briefly rinsed, fixed in 10% formalin and stained with Gram's crystal violet solution (Fluka, Stockholm, Sweden). Cells from a minimum of three high power fields per insert were counted under light microscopy (Olympus, Solna, Sweden). Statistical comparisons between migration of individual cell clones were carried out by ANOVA with Bonferroni post-hoc correction.

For matrix invasion assays, the haptotaxis assay above was modified such that instead of fibronectin, the basement membrane matrix mixture Matrigel (BD Biosciences, Bedford, MA) was used to cover the upper side of cell culture inserts with 8 µm pores. Separate clones of Mock and Tensin3-transfected 293 cells were seeded into the chamber interiors in medium containing 0.5% serum, whilst the wells of 24-well plates contained complete growth medium, thereby creating a serum gradient. Duplicate inserts were used for each separate cell line analyzed. Cells were allowed to invade the matrix and migrate through to the other side of the membrane over 30 h at 37°C. Afterwards, cells were fixed, stained and counted as described above.

### Knockdown of Tensin3 by siRNA silencing

**Figure 1:**
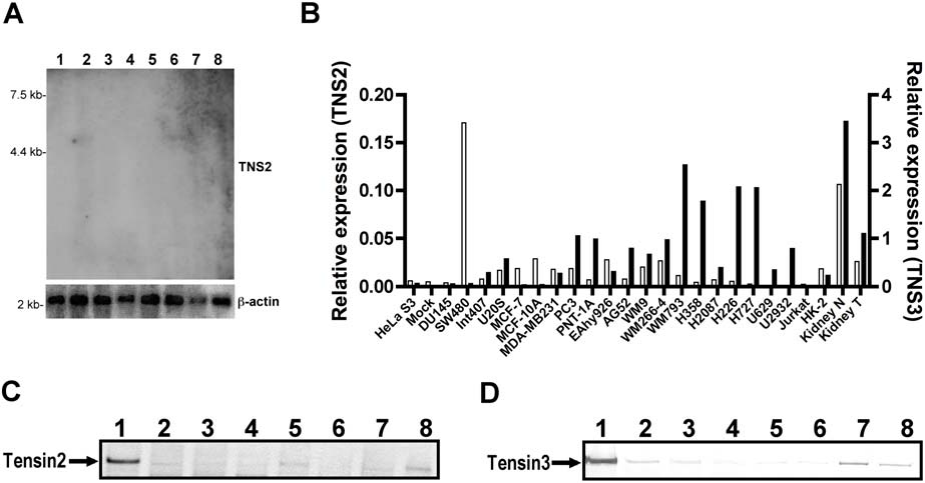
Absence of expression of Tensin2 and Tensin3 at both mRNA and protein levels in several different human cancer cell lines. (A) Northern blot analysis of Tensin2 (TNS2) mRNA in human cancer cell lines. A human cancer cell lines MTN blot was probed at high stringency with radiolabeled cDNA specific for Tensin2. Lanes are as follows: 1, promyelocytic leukemia HL-60; 2, HeLa S3; 3, chronic myelogenous leukemia K-562; 4, lymphoblastic leukemia MOLT-4; 5, Burkitt's lymphoma Raji; 6, colorectal adenocarcinoma SW480; 7, lung carcinoma A549 and 8, melanoma G-361. The same membrane was also probed for human β-actin (lowest panel) to show equal RNA loading. A representative blot is shown of two independent hybridizations. (B) mRNA expressions of Tensin2 and Tensin3 analyzed by qRT-PCR. A panel of human cancer cell lines as well as human normal epithelial and endothelial cell lines were screened for Tensin2 (TNS2) expression (left axis, empty bars) and Tensin3 (TNS3) expression (right axis, black bars). Results are presented as relative expression given after normalization of gene of interest to reference gene *GAPDH* (ng gene of interest/ng reference gene). (C, D) Expression of Tensin2 and Tensin3 proteins in human cancer cell lines. Whole cell lysates were subjected to 8% SDS-PAGE and western blot detection of Tensin2 and Tensin3 proteins. Lanes are as follows: 1, HEK 293 cells stably transfected with Tensin2 (C); 1, HEK 293 cells stably transfected with Tensin3 (D) 2, renal carcinoma SKRC-52; 3, HeLa S3; 4, colorectal adenocarcinoma SW480; 5, breast adenocarcinoma MCF-7; 6, prostate carcinoma DU145; 7, melanoma WM793; 8, lung carcinoma H727.

The melanoma cell line WM793 was selected for Tensin3 gene silencing as it expressed the highest amounts of Tensin3 out of all the cancer cell lines tested (Figure 1B). Cells were seeded in 6-well plates 24 h prior to siRNA transfection. Cells were separately transfected with 5 nM each of three different Tensin3 siRNA constructs, siRNA I, II (ON-TARGET plus siRNA Reagents; Dharmacon, Täby, Sweden) and III (HP validated siRNA; Qiagen, Solna, Sweden). Negative controls included transfection mixture only and a non-silencing siRNA that was shown to have little effect on global gene expression (AllStars; Qiagen, Solna, Sweden). The siRNA was mixed with Lipofectamine 2000 and OptiMEM I serum free medium (Invitrogen, Lidingö, Sweden), and the tranfection mixture was incubated with cells for 24 h. After this period, cells were trypsinized, counted and subjected to a migration/haptotaxis assay exactly as described above, except that 0.5×10^5^ WM793 cells per insert were used, and migration was allowed to occur over 16 h.

### Statistical analysis

All clinical data were analyzed using the SPSS software package (version 16.0.2 for Macintosh; SPSS Institute, Chicago, IL). Mean differences of non-normally distributed data were analyzed by the Mann-Whitney test. The Kruskal-Wallis test was used for comparison between more than two groups. Correlations were assessed by Spearman rank correlation test. Survival curves were evaluated with the Kaplan-Meier method and survival times were compared using the log-rank test.

In cell proliferation, migration and invasion assays, statistical comparisons between different cell clones or treatments were carried out by ANOVA with Bonferroni post-hoc correction. All statistical tests were two-sided and a *P* value of less than 0.05 was considered to be statistically significant.

## Results

### Tensin expression is largely absent in human cancer cell lines

We first screened a panel of human cancer cell lines for Tensin expression. A selection of different human cancer cell lines was screened by Northern blot analysis for expression of Tensin2 mRNA. The expected band at 5 kb for Tensin2, as previously reported in human organs [1], was barely detectable in only 2 out of 8 cell lines: HeLa and SW480 (Figure 1A). We went on to perform quantitative RT-PCR to further investigate mRNA expression levels of Tensin2 and Tensin3 in a larger selection of human cancer cell lines and compared them directly to RNA extracted from whole human kidney tissue extracts. As shown in Figure 1B, Tensin2 mRNA was absent in the majority of cell lines. Only the colorectal adenocarcinoma cell line SW480 expressed Tensin2 mRNA at detectable levels, which was also seen in the Northern blot (Figure 1A). Significantly, both Tensin2 and Tensin3 expression were lower in a single RCC patient's kidney tumor extract as compare to its adjacent normal counterpart (matched). The results show that Tensin3 expression is higher and broader than that of Tensin2 amongst the investigated cell lines, but that both genes appear to be downregulated in RCC. We also examined Tensin1 and Tensin4 mRNA in the same panel of samples and found expression of these Tensins to be largely undetectable or totally absent (data not shown).

We performed immunoblot to analyze the protein expression of Tensin2 and Tensin3 (Figure 1C, D). In keeping with the mRNA results, expression of Tensin2 at the protein level (molecular weight 160 kDa) was hardly detected in human cancer cell lines, whereas Tensin3 (180 kDa) expression was detectable at least in two cell lines: melanoma cell line WM793 and lung carcinoma line H727. Together these results show that expression of all Tensins is largely absent in human cancer cell lines as well as being downregulated in human kidney tumors.

### Tensins 1–4 mRNA levels are significantly lower in renal cell carcinoma versus normal kidney tissue

We applied multiplex one-step qRT-PCR to evaluate mRNA expression levels of all four Tensins in this clinical study of total RNA extracted from 223 RCC and 48 normal kidney cortex tissue samples. The clinical and pathological characteristics of the RCC patients in this study are shown in Table 1. The normal samples were obtained from matched tumor sources and the distribution of tumor types amongst these was similar to that in the overall population, ie. 75% were of clear cell type (Table 1). From a screen of 11 candidate housekeeping genes (Applera Sweden, Stockholm, Sweden), we empirically determined the *B2M* gene to be the most consistent and robust internal standard to use for human kidney tissue (data not shown).

**Figure 2:**
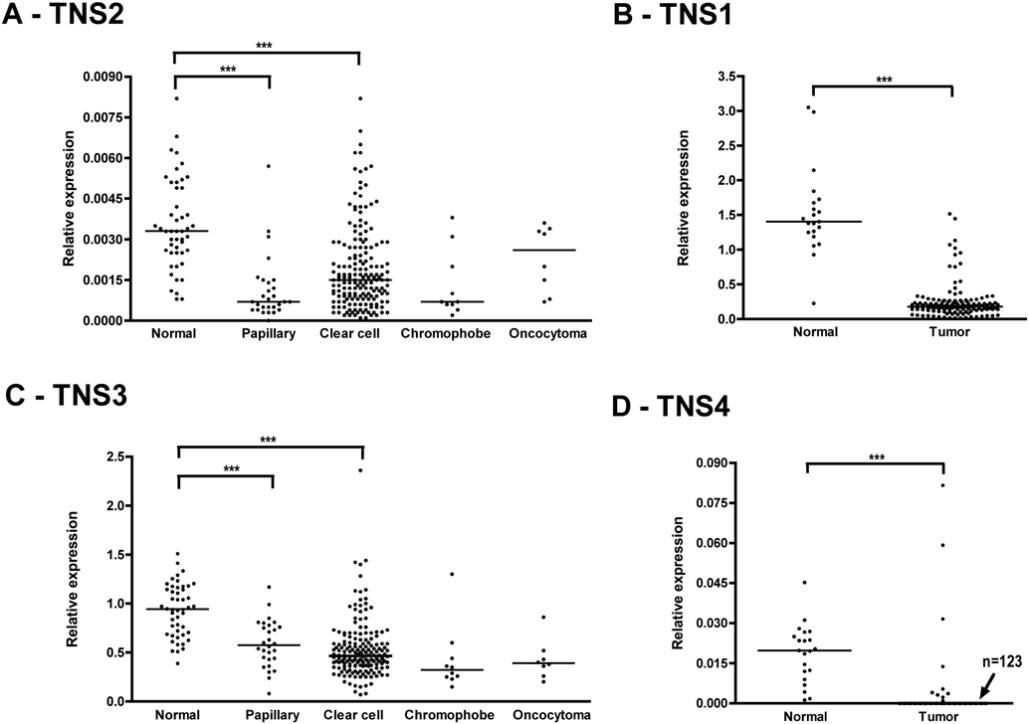
Downregulation of Tensins 1–4 mRNA expression in human kidney cancer. (A,C) mRNA levels of Tensin2 (TNS2) and Tensin3 (TNS3), respectively in 223 RCC patients grouped according to tumor type, vs normal kidney cortex samples from 48 patients. Results are presented as relative expression (ng Tensin2/ng B2M and ng Tensin3/ng B2M, respectively). Tensin2 and Tensin3 expression in relation to tumor type as well in control group are shown along with the median value. ^***^ *P*<0.001 normal vs. papillary and for normal vs. ccRCC. (B,D) mRNA levels of Tensin1 (TNS1) and Tensin4 (TNS4), respectively in 134 RCC cases (tumor) and 21 kidney cortex samples (normal) after normalization to reference gene (relative expression). Medians are shown for both Tensin1 and Tensin4 expression in normal and tumor samples. In panel D, the value n = 123 is indicated as the number of tumor samples with no Tensin4 expression. ^***^ *P*<0.001 normal vs. tumor.

As shown in Figure 2A, mRNA expression of Tensin2 from 223 RCC extracts was significantly lower than in extracts from normal kidney cortex (48 cases; *p*<0.001). The results are largely reflected from ccRCC, which represented the majority of cases; however the lower levels in pRCC were also of high significance (*p*<0.001). Furthermore, analysis of only the subgroup of tumor material that had matched normal counterparts yielded also a significantly lower Tensin2 and -3 expression in the tumor tissues (n = 48; *p*<0.001). In addition, there was also a statistically significant drop in Tensin1 expression in tumor samples (n = 134) compared to normals (n = 21) (Figure 2B). A similar and highly significant reduction in Tensin3 mRNA expression was also observed (Figure 2C). Notably, Tensins -1, -2 and -3 expression was detected in all samples measured. In contrast, Tensin4 expression was largely absent in the majority of RCC samples measured (n = 134), whilst it was present in all normal kidney cortex samples (n = 21) (Figure 2D). These findings, in conjunction with those described above, show that a general drop in the expression of Tensins occurs in RCC and could therefore be favorable to the development of RCC tumors.

### Correlation between Tensin expression and clinical variables in RCC patients

We performed correlation analyses to evaluate a potential relation between Tensin mRNA expression in RCCs and the clinical characteristics of the patients. The most relevant correlations are summarized in Table 2. Firstly, mRNA expression of Tensins 1–3 in tumors correlated positively with each other, though not with Tensin4 as it was largely undetectable in RCC samples. No significant correlation was apparent between Tensins mRNA expression levels and clinical variables as tumor size, tumor venous infiltration, regional lymph node infiltration, age or gender. However, there was a notable negative correlation between tumor grade and both Tensin1 and Tensin3 expression in tumors, ie. low expression correlated with a high tumor grade. Similarly, despite a lack of significance, a trend towards a negative correlation was apparent for tumor grade vs Tensin2 and Tensin4 mRNA expression in tumors.

**Table 2:** Correlation between mRNA expressions of Tensins 1–4 (TNS1-4) in RCC patients and various clinical variables.

	TNS1 mRNA in tumor	TNS2 mRNA in tumor	TNS3 mRNA in tumor	TNS4 mRNA in tumor
Tumor grade	(1) **−0.196**	−0.107	**−0.115**	−0.131
	(2) 0.026*	0.116	0.023*	0.136
	(3) 129	215	215	131
Tumor size	0.089	−0.063	−0.069	−0.005
	0.308	0.350	0.304	0.952
	134	223	223	134
Venous invasion	0.001	−0.055	−0.064	−0.102
	0.994	0.417	0.341	0.242
	134	223	223	134
Regional lymph node involvement	−0.022	−0.138	0.016	0.281
	0.888	0.229	0.892	0.068
	44	78	78	43
TNS1 mRNA in tumor	-	**0.555**	**0.342**	0.074
	-	<0.001***	<0.001***	0.529
	-	134	134	74
TNS2 mRNA in tumor	**0.555**	-	**0.515**	**0.199**
	<0.001***	-	<0.001***	0.021*
	134	-	223	133
TNS3 mRNA in tumor	**0.342**	**0.515**	-	0.075
	<0.001***	<0.001***	-	0.394
	134	223	-	133
TNS4 mRNA in tumor	0.074	**0.199**	0.075	-
	0.529	0.021*	0.394	-
	74	133	133	-

(1) *r* value, significant values in **bold**; (2) *P* value; (3) number of cases; *P<0.05; ***P<0.001.

### Immunohistochemical characterization of Tensin3 expression in kidney tumor tissue microarrays (TMA)

**Figure 3:**
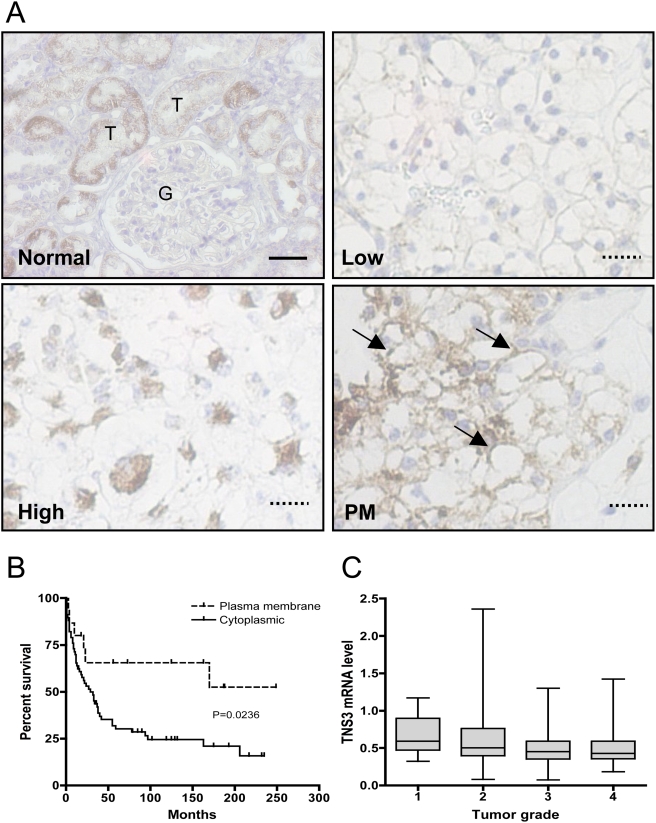
Presence of immunohistochemical staining of Tensin3 at the plasma membrane correlates with increased patient survival. (A) Photomicrographs of normal human kidney (upper left) and ccRCC sections (remaining; 3× higher magnification), showing differential staining patterns for Tensin3: low (upper right), high (lower left) and additionally at the plasma membrane (PM; indicated by arrows; lower right). In normal human kidney, Tensin3 staining is present almost exclusively in proximal tubular epithelium (T) and is characterized by a granular brown staining within the cytosol, while being absent in the glomerulus (G). Whole scale bar represents 15 µm, while dotted scale bars are 5 µm. (B) Kaplan-Meier plot illustrating survival curves of 88 patients with clear cell RCC tumors displaying Tensin3 staining in the cytoplasm (n = 72) and also at the plasma membrane (PM, n = 16). (C) A box plot of Tensin3 (TNS3) mRNA level in relation to tumor grade (n = 215). ^*^ *P*<0.05, statistical significance in Tensin3 expression over range of tumor grades (Kruskal-Wallis test).

Owing to its higher and more prevalent expression in the kidney, we investigated Tensin3 expression additionally by immunohistochemical analysis of RCC sections in TMA format. We found that Tensin3 protein was expressed in the normal human kidney cortex and is clearly restricted to proximal tubular epithelial cells, whilst being absent or negligible in glomeruli and podocytes (Figure 3A). This was in contrast to Tensin2, which appeared to be present primarily in podocytes in the mouse [15]. Positive immunohistochemical staining for Tensin3 in RCC tissue sections was found in the cytoplasm and at varying intensities. There was no Tensin3 expression in the fibrovascular stroma or endothelial cells of tumour vessels. Positive staining was typified by a granular, dotted brown staining throughout the cytosol. Out of a total of 148 patients with RCC tumor sections analyzed, 61 (41%) displayed either no Tensin3 staining or very weak cytoplasmic staining. In contrast, a subset of sections (16) showed an additional strong and defined Tensin3 immunoreactivity at the plasma membrane. Separating the data according to tumor type, no significant correlation was apparent between Tensin3 staining intensity and patient survival (data not shown). However, in ccRCC patients, presence of Tensin3 staining at the plasma membrane of positive cells (PM) conferred a statistically significant survival advantage over those ccRCC tumors with only cytoplasmic expression (Figure 3B). Moreover, Tensin3 PM staining in tumor TMAs tended to correlate negatively with the kidney tumor stage according to the TNM system (*P* = 0.068), as well as tumor size (*P* = 0.125; data not shown). In keeping with the TMA results, tumor Tensin3 mRNA expression in RCC patients was significantly correlated with tumor grade in a negative manner, where decreased Tensin3 mRNA levels were associated with a higher tumor grade (n = 215; *P* = 0.0471; Figure 3C).

### Overexpression of Tensin3 in human kidney cells does not affect proliferation but markedly reduces cell migration and invasion

**Figure 4:**
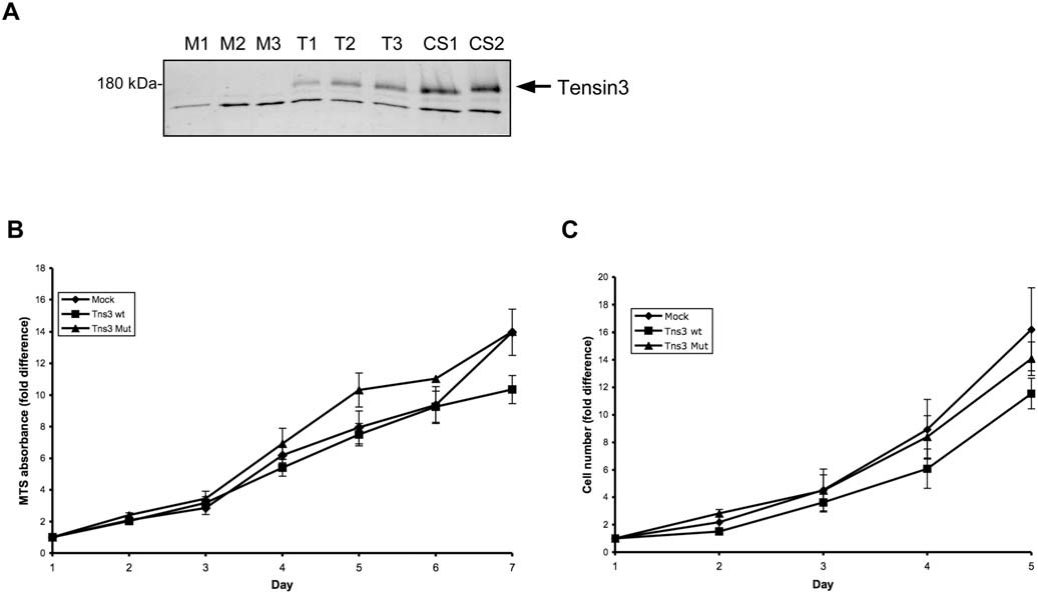
Stable expression of Tensin3 in HEK 293 cells and effect on proliferation. (A) Western blot analysis of recombinant Tensin3 expression in individual stable HEK 293 cell clones. Cell lysate proteins were separated by 5% SDS-PAGE, followed by western blotting with an anti-human Tensin3 antibody and chemiluminescent development. Three clones of mock cells (M1-3), 3 of wildtype Tensin3 cells (T1-3) and 2 of mutant Tensin3 cells (CS1-2) were analyzed. The upper bands correspond to expected size of Tensin3; lower bands are non-specific immunoreactivity, which is present in all cell extracts. (B) Proliferation of stable Tensin3-expressing cells by measurement of viable cell number through mitochondrial activity. Equal numbers of mock cells, wildtype Tensin3 cells (Tns3 wt) and Tensin3 PTPase mutant cells (Tns3 Mut) were incubated in medium containing 5% fetal calf serum. On days 1–7, conversion of MTS into soluble formazan during 3 h by viable cells was measured spectrophotometrically at 490 nm. Points represent the fold difference in Abs_490_ compared to that on day 1 (quadruplicate wells per measurement). Results are mean±SEM (n = 5 separate experiments, pooling results from 3 mock cell clones vs 3 Tns3 wt cell clones vs 2 Tns3 Mut cell clones). All cell types grew at similar rates, and there was no statistical difference between cell types on each day (ANOVA). (C) Growth curves of stable Tensin3-expressing cells by measurement of absolute cell number. Equal numbers of mock cells, wildtype Tensin3 cells (Tns3 wt) and Tensin3 PTPase mutant cells (Tns3 Mut) were incubated in medium containing 5% fetal calf serum. On days 1–5, cells were trypisinized and counted on a cell counting chamber. Points represent the fold difference in cell number compared to that on day 1. Results are mean±SEM from two pooled separate experiments, comparing 3 mock cell clones vs 3 Tns3 wt cell clones vs 2 Tns3 Mut cell clones. All cell types grew at similar rates, and there was no statistical difference between cell types on each day (ANOVA).

**Figure 5:**
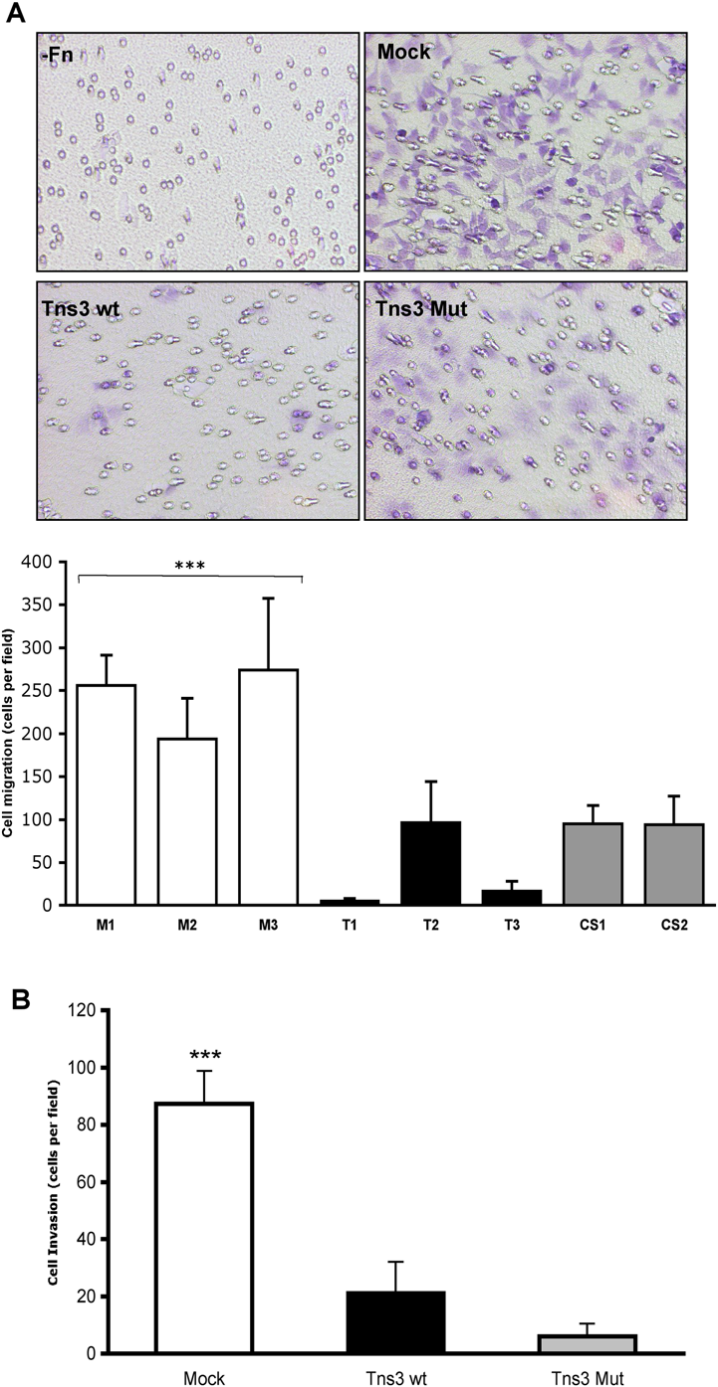
Tensin3 expression inhibits haptotactic HEK 293 cell migration as well as their invasion through extracellular matrix. (A) Migration assay. Equal numbers of HEK 293 cells stably transfected as mock, wildtype Tensin3 (Tns3 wt) and PTPase mutant Tensin3 (Tns3 Mut) were allowed to migrate towards fibronectin in cell culture inserts for 18 h at 37°C. The upper panel shows light micrographs of the undersides of the chambers showing cells that had migrated to the lower side of the membranes and stained with crystal violet. Chambers shown are those with mock cells but no fibronectin (−Fn, upper left; only pores are visible), mock cells (upper right), Tns3 wt (lower left), and Tns3 Mut (lower right). The lower panel is a quantitative representation of the migration of individual stable transfected cell clones: mock cells (M1-3), wildtype Tensin3 cells (T1-3) and Tensin3 PTPase mutant cells (CS1-2) (characterized in Figure 4A). Each bar represents mean±SD migrated cells (n = 6 fields counted per insert, duplicate inserts per clone). *** denotes *P*<0.001 for each mock cell clone vs each of the Tns3 clones (AVONA with Bonferroni adjustment). (B) Matrigel invasion assay. Equal numbers of HEK 293 cells stably transfected as mock, wildtype Tensin3 (Tns3 wt) and PTPase mutant Tensin3 (Tns3 Mut) were allowed to migrate through a Matrigel layer on top of a cell culture insert membrane over 30 h at 37°C. As for migration assay, cells on the lower side of the membranes at 30 h were fixed, stained with crystal violet and counted. Bars represent mean±SEM number of invaded cells (n = 3 cell clones per type (2 for Tns3 Mut); duplicate inserts per clone; 6 fields counted per insert). *** denotes *P*<0.001 for mock cells vs Tns3 wt and Tns3 Mut cells (ANOVA with Bonferroni adjustment).

Individual clones of HEK 293 cells stably transfected with empty vector (mock), wildtype Tensin3 or PTPase mutant Tensin3 were generated. These were first verified for expression (or lack thereof) of Tensin3 by western blot (Figure 4A). Stable expression of Tensin3 in these cells did not affect their proliferation rates in serum as compared to mock-transfected cells (Figures 4B and 4C). However, Tensin3 expression caused greatly reduced rates of both haptotactic migration (towards an extracellular matrix gradient) and invasion of basement membrane matrix, as compared with mock cells (Figure 5). After 18 h of incubation, no cell migration through 8 µm pores had occurred in the absence of a fibronectin layer on the other side of the membrane (Figure 5A). However, in the presence of a fibronectin gradient, each clone of Tensin3 wt and Tensin3 mutant cells displayed a migration rate at least 50% lower than that of the mock cell clones (Figure 5A). Therefore, Tensin3 inhibited cell migration and moreover, a potential phosphatase-dead version of Tensin3 did not hinder Tensin3 from this suppressive effect. In addition, Tensin3 expression also inhibited the capacity of 293 cells to invade through a basement membrane matrix towards a serum gradient, which was also apparent in cells expressing mutant Tensin3 (Figure 5B).

### Gene silencing of endogenous Tensin3 in human cancer cells by siRNA increases their migration

**Figure 6:**
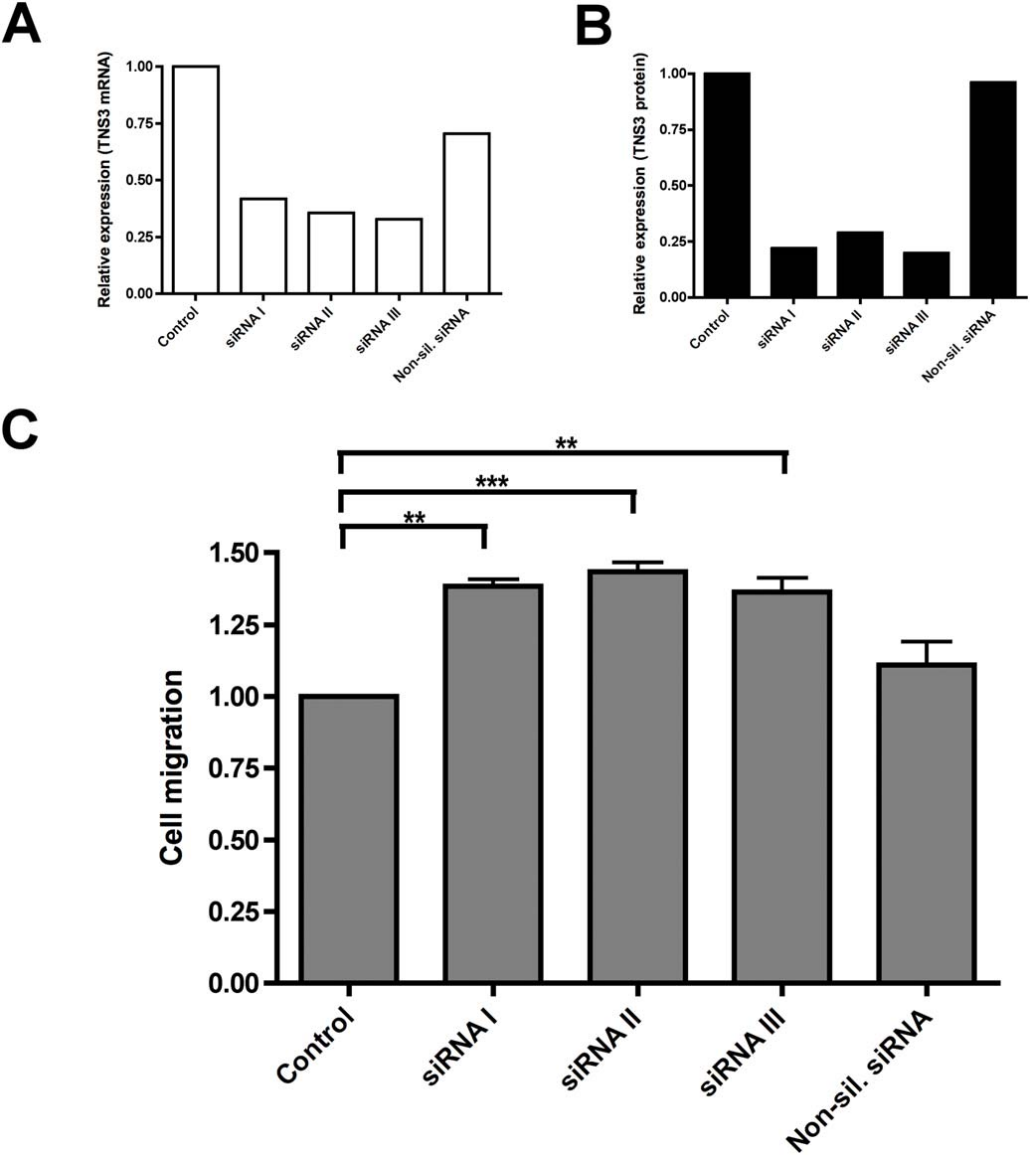
siRNA knockdown of endogenously expressed Tensin3 in human melanoma cells increases cell migration. Endogenous Tensin3 expression was knocked down over 24 h in human melanoma cells WM793 using three different Tensin3 siRNA constructs (siRNA I, II, III), as well as non-silencing siRNA and transfection mixture only (negative controls). (A) mRNA level of Tensin3 in WM793 cells after 24 h gene silencing, analyzed by qRT-PCR. Results of gene expression were normalized to endogenous control gene (*B2M*) and are presented as Tensin3 (TNS3) expression relative to non-silenced control (transfection mix only; first bar). Bars shown are means of duplicate determinations from a representative experiment of four. (B) Protein level of Tensin3 (TNS3) in WM793 cells after 24 h gene silencing, analyzed by western blot. Whole cell lysates were subjected to 8% SDS-PAGE and western blot detection with anti-Tensin3 antibody. Band intensities were quantified by densitometry and normalized to GAPDH protein level on the same blot. Bars are Tensin3 protein expression relative to non-silenced control (transfection mix only; first bar). (C) Migration of WM793 cells over 16 h, treated with different siRNA constructs as well as negative controls (transfection mix only, and non-silencing siRNA). Each bar represents mean±SEM migrated cells expressed as fold difference relative to negative control cells (transfection mix only) (n = 3 separate experiments, duplicate inserts). ***P<0.001 control vs. Tensin3 siRNA II; **P<0.01 control vs. Tensin3 siRNA I and III; no significant difference for control v non-silencing siRNA (ANOVA with Bonferroni adjustment).

Due to fact that overexpression of Tensin3 in 293 cells markedly reduced cell migration, we performed siRNA knockdown experiments to investigate the role of endogenous Tensin3 in migration of a human primary cancer cell line. The human melanoma cancer cell line WM793 was chosen due to its high endogenous expression of Tensin3 relative to other cancer cell lines (Figure 1B). Converse to the inhibitory effect of overexpressing Tensin3 in normal cells, WM793 cells showed an increased migration when treated with specific siRNA for Tensin3 (Figure 6). We utilized three separate, validated siRNA constructs to knockdown endogenous Tensin3, and all three were effective in reducing Tensin3 mRNA expression by at least 50% in comparison to negative controls (Figure 6A). This knockdown of Tensin3 by all three siRNAs was also observed at the protein level by western blotting (Figure 6B). The silencing of endogenous Tensin3 by each of the three siRNAs caused a significant increase (30%) in haptotactic migration of WM793 cells as compared to non-silenced controls (Figure 6C). Thus, suppression of endogenous Tensin3 expression enhances cancer cell migration, in keeping with the converse effects seen with ectopic expression.

## Discussion

Despite advances in research that have identified molecular triggers for RCC, such as dysfunction of the VHL tumor suppressor gene, the picture is far from complete and there is a need to further define the pathogenesis of RCC and identify potential targets for therapy. The predicted five-year survival rate for RCC patients at all stages is 67% [13], 47% in patients with renal involvement [24], and only 10% in patients with stage IV disease (metastasis) [13]. Furthermore, most of advanced RCC patients do not respond to standard cytokine therapy [25] and the novel tyrosine kinase inhibitors generally only stabilize the disease [26]. This therefore makes urgent the need for novel therapies particularly aimed at treating metastatic disease.

Today there exist dozens of intracellular molecules, the loss of which are associated with the neoplastic state in cells. This study is the first comprehensive investigation of the Tensin family of intracellular proteins in human kidney cancer, and moreover in human clinical disease. We have studied all four members of the Tensin family and, in keeping with a proposed tumor/metastasis suppressor role, found them all to be reduced in expression in RCC. Tensins -2 and -3 were absent or barely expressed in a variety of human cancer cell lines. Furthermore, a preferential plasma membrane expression of Tensin3 was correlated with greater survival. In addition, recombinant Tensin3 expression in human kidney cells caused a greatly reduced haptotactic cell migration and matrix invasion, indicating a role for Tensins in control of cell motility.

Our quantitative RT-PCR, western blot and immunohistochemistry results indicate that Tensin3 and Tensin1 are likely the most prevalent Tensins in the human kidney. Tensin3 is expressed preferentially in renal proximal tubular epithelium, while Tensin2 may also be present in podocytes [15], although Tensin2 is expressed at a lower level in total. All four Tensins were found to be highly significantly lower or absent in RCC tissue versus matched normal kidney cortex tissue. Notably, Tensin3 gene expression was significantly negatively correlated with tumor grade. The significance of the expression of Tensin3 and its loss in tumor development has been reported for breast cancer cells, where Tensin3 downregulation was coupled to lesser cytoskeletal stability and consequently greater motility [9]. Downregulation of Tensin3 in thyroid tumors was also shown [27]. Tensin2 downregulation in tumourigenesis has been observed in hepatocellular carcinoma [28] and uterine carcinoma [29]. This is the first observation of a downregulation of Tensin1 in human cancer, and may also be relevant, as it has previously been shown that genetic deletion of Tensin1 in mice causes renal dysfunction [17]. In the case of Tensin4, this Tensin has been observed at both higher [9], [30], [31] and lower [8] levels in different clinical tumor samples. One study has reported results indicating that Tensin4 may compete with Tensin3 in sites where it is expressed strongly enough [9]. However, in our study we found Tensin4 to be the least expressed of all the Tensins in human kidney, and it was in fact absent in most RCCs analyzed. Moreover, all the Tensins are able to interact with DLC-1 tumor suppressor protein, and may therefore enable its localization and proper function [10]–[12]. Therefore, all together these results indicate that a general loss of Tensins may be advantageous to the development or spread of tumors.

The immunohistochemical analysis of Tensin3 protein expression and localization in RCC, using TMAs, showed an interesting negative correlation between its plasma membrane localization and patient survival. This indicates that in ccRCC patients where tumor cells have Tensin3 present at the plasma membrane have a better survival prognosis than ccRCC patients lacking Tensin3 or expressing it only within the cytoplasm. In keeping with data supporting the necessity of Tensin3 for cytoskeletal stability and organization [9], observation of Tensin3 at the cell periphery may indicate that a tumor cell is more static and thus less likely to be motile and metastasize.

We investigated the biological role of Tensin3 in various cell models. Normal kidney cells were stably transfected with both wild type Tensin3 as well as a Tensin3 mutant that would potentially abolish any phosphatase activity. Stable expression of Tensin3 did not affect cell viability and proliferation; however, it affected both cell migration and invasion negatively. Tensin3-expressing cells exhibited a markedly reduced migration towards a fibronectin gradient as compared to mock-transfected cells. Furthermore, expression of a putative phosphatase-dead Tensin3 also inhibited migration to the same degree, indicating that the effect is independent of a phosphatase activity in the protein. In addition, Tensin3 also markedly reduced invasion of these cells into a basement membrane matrix. Moreover, we observed the opposite effect, i.e increased cell migration, using siRNA to knock down endogenous Tensin3 in human cancer cells. These results demonstrate that Tensin3 stabilizes cells and prevents their uncontrolled migration under normal conditions. However, loss of Tensin3, as occurs in e.g. RCC and other cancers, may underlie the increased cell motility in cancer cells and their propensity to subsequently metastasize, as has been alluded to previously [9]. Furthermore, any putative PTEN-like phosphatase activity in Tensin3 is not necessary for this potential anti-metastatic capacity of Tensin3. We have previously characterized the function of Tensin2 in kidney cells and found Tensin2 to suppress the Akt signaling pathway as well as inhibit cell proliferation, survival and migration [5]. Moreover, Tensin1 null cells have also been shown to exhibit impaired migration [32]. Taken together, it is therefore likely that the cell motility modifying capacity of all Tensin family members is a common feature relying on their integrin/cytoskeletal interactions. In contrast, Akt pathway suppression may be unique amongst those members of the Tensin family that may have a lipid phosphatase activity, ie potentially only Tensins -2 and -3, and may be linked to cell survival and proliferation.

Much still remains to be uncovered as to the range of functions various tumor suppressors play. Understanding the biological characteristics of individual tumor suppressors and their relation to specific diseases will certainly provide new avenues in the future for screening, diagnosis and treatment. Studies on Tensins so far including the present one indicate that the Tensins represent a unique family of intracellular proteins that are able to link the extracellular matrix, via integrins and cell surface receptors, to the cytoskeleton and thereby control cytoskeletal organization and consequent migratory capacity of cells. This feature may be necessary for maintenance of normal cell architecture, and when disrupted, for example after long-term stimulation by growth factors, would result in cellular instability and greater motility. The ensuing cell behavior would thus be expected to favor the metastatic process.

We have shown that the expression of all Tensins is downregulated inRCC, and therefore analyzing gene expression and/or subcellular localization of one or more of the Tensins could be useful complements for diagnostic and prognostic estimations. The present study further suggests the potential value of therapies aimed at potentiating or upregulating the Tensins as an anti-metastatic strategy. This is already a prospect with existing agents under trial, such as the cancer chemopreventative polyphenol resveratrol, which was shown to markedly upregulate Tensin1 in different cancer cell lines [33].

## Supporting Information

Figure S1 Anti-Tensin3 antibody. Polyclonal anti-Tensin3 (Tns3) antibody was verified for specificity by antigen blocking. Antibody was incubated overnight at the optimal dilution of 1∶300 either alone (A), or together with the peptide antigen it was raised against (Ag; B), or with the recombinant phosphatase domain of Tns3 (PTPase, negative control, C). Potential immune complexes were removed by centrifugation, followed by immunohistochemical testing of the mixtures on normal human kidney section as described (Methods). Bar represents 15 micrometers. Note that Tns3 immunoreactivity is completely blocked by the relevant antigen (B) but not by an irrelevant antigen from the same protein (C).
(6.65 MB PPT)
