# Supplementary material for: Tensin3 Is a Negative Regulator of Cell Migration and All Four Tensin Family Members Are Downregulated in Human Kidney Cancer
Source: PLoS One. 2009 Feb 4;4(2):e4350. doi: 10.1371/journal.pone.0004350 (PMC2632886; doi:10.1371/journal.pone.0004350)
Supplement: Figure S1 — Anti-Tensin3 antibody. Polyclonal anti-Tensin3 (Tns3) antibody was verified for specificity by antigen blocking. Antibody was incubated overnight at the optimal dilution of 1∶300 either alone (A), or together with the peptide antigen it was raised against (Ag; B), or with the recombinant phosphatase domain of Tns3 (PTPase, negative control, C). Potential immune complexes were removed by centrifugation, followed by immunohistochemical testing of the mixtures on normal human kidney section as described (Methods). Bar represents 15 micrometers. Note that Tns3 immunoreactivity is completely blocked by the relevant antigen (B) but not by an irrelevant antigen from the same protein (C). (6.65 MB PPT) [file pone.0004350.s001.ppt]

## Slide 1
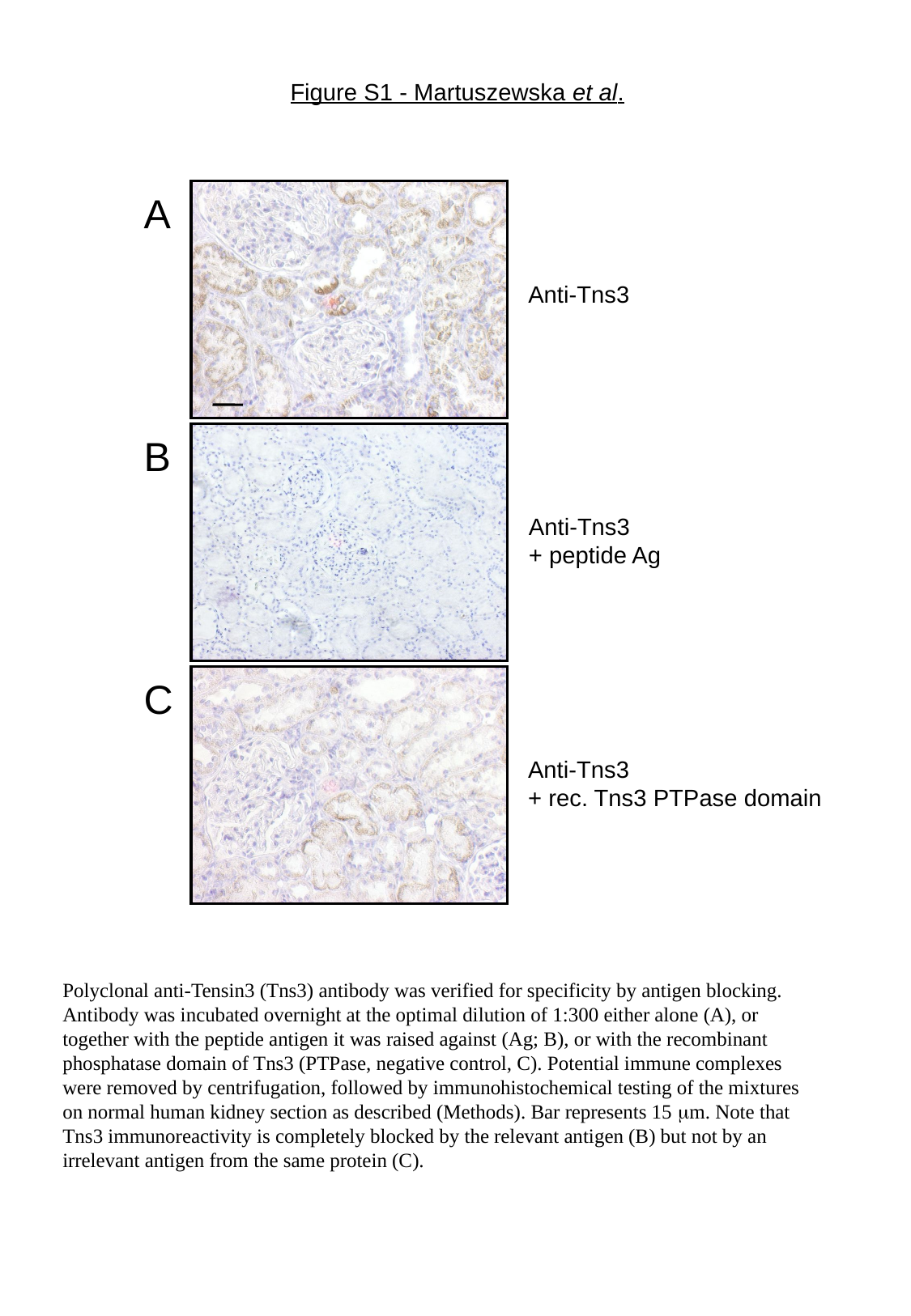

Figure S1 - Martuszewska et al.
A
Anti-Tns3
B
Anti-Tns3
+ peptide Ag
C
Anti-Tns3
+ rec. Tns3 PTPase domain
Polyclonal anti-Tensin3 (Tns3) antibody was verified for specificity by antigen blocking. Antibody was incubated overnight at the optimal dilution of 1:300 either alone (A), or together with the peptide antigen it was raised against (Ag; B), or with the recombinant phosphatase domain of Tns3 (PTPase, negative control, C). Potential immune complexes were removed by centrifugation, followed by immunohistochemical testing of the mixtures on normal human kidney section as described (Methods). Bar represents 15 μm. Note that Tns3 immunoreactivity is completely blocked by the relevant antigen (B) but not by an irrelevant antigen from the same protein (C).
